# Upper Gastrointestinal Bleeding Secondary to Duodenal Wall Perforation by Inferior Vena Cava Filter: A Rare Clinical Presentation

**DOI:** 10.7759/cureus.48448

**Published:** 2023-11-07

**Authors:** Hazem Abosheaishaa, Utsow Saha, Omar Abdelhalim, Nuha Al-Howthi, Ahmed Elhawary, Mohammed Abusuliman

**Affiliations:** 1 Internal Medicine, Icahn School of Medicine at Mount Sinai, Queens Hospital Center, New York, USA; 2 Internal Medicine/Gastroenterology, Cairo University, Cairo, EGY; 3 Medical Education, Enam Medical College and Hospital, Rangpur, BGD; 4 Medicine, Icahn School of Medicine at Mount Sinai, Queens Hospital Center, New York, USA; 5 Internal Medicine, Henry Ford Health System, Detroit, USA

**Keywords:** esophagogastroduodenoscopy, ivc filter, duodenal wall perforation, endoscopy, upper gastrointestinal bleeding

## Abstract

Patients with venous thromboembolism (VTE) frequently employ inferior vena cava (IVC) filters to keep them from getting pulmonary embolisms. Even though they are usually thought to be safe, there can be complications during or after their placement. IVC filter perforation into adjacent structures, such as the duodenum, is an uncommon but potentially serious complication. We present a case of a 62-year-old female with a past medical history of recurrent deep vein thrombosis (DVTs) and pulmonary embolism who presented with dizziness and dyspnea due to gastrointestinal (GI) bleeding, resulting in anemia. Esophagogastroduodenoscopy (EGD) was done and revealed a metallic object extending into the duodenum, identified as the IVC filter.

## Introduction

For a long time, vena cava filters have been used as a way to treat venous thromboembolism (VTE). Most of the time, they are put in the inferior vena cava (IVC), but sometimes they are put in the superior vena cava (SVC). These filters are very important for preventing pulmonary embolisms [1]. IVC filters are usually put in place in an interventional room with the help of fluoroscopy to make sure they are put in the right place. You can also use methods like fluoroscopy, transabdominal ultrasound, or intravascular ultrasound to put these filters at the bedside. For vena cava filters to work and to keep problems from happening, doctors must be able to tell the difference between normal and abnormal vena cava structures [2]. IVC filters can cause complications during or after the placement process. Problems during the placement procedure can be caused by technical issues or side effects of the medicine, or they can happen later on because of problems with the access site or problems with the filter itself such as duodenal and aorta perforations which can happen when an IVC filter is put in. These can be painful and cause pain in the abdomen, also bleeding from late consequences like duodenocaval fistula can be life-threatening [3].

## Case presentation

**Figure 1:**
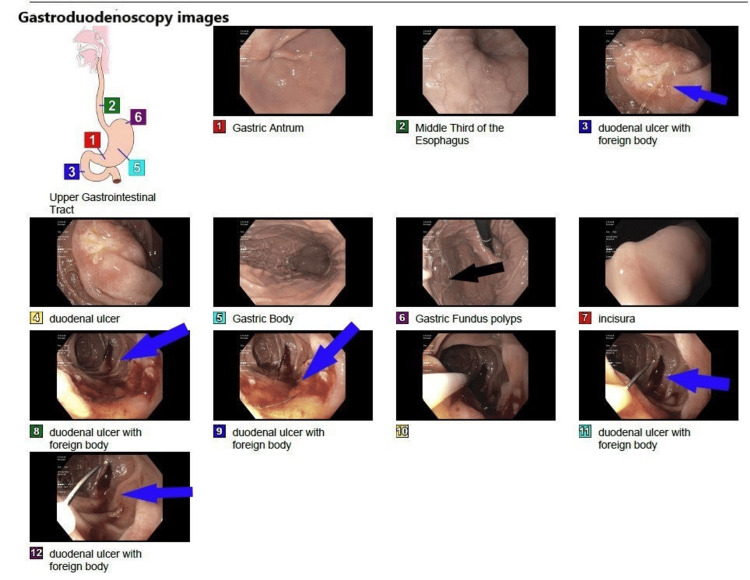
Endoscopy showing fundal gastric polyps (panel 6, black arrow) and duodenal ulcer with IVC filter invading the duodenal wall (panels 3, 8, 9, 11, and 12 blue arrows)

A 62-year-old female patient was admitted to the hospital with the impression of abdominal pain, worsening dizziness, and dyspnea due to blood loss anemia from GI bleeding with initial hemoglobin of 8.8 mg/dl down trending from 10.4 mg/dl. Her past medical history is significant for recurrent lower extremity DVT and pulmonary embolism (PE) with IVC filter placement. On presentation, vitals were stable with a negative physical exam except for epigastric tenderness without guarding or rigidity. A negative CT angiography finding ruled out the suspicion of pulmonary embolism as the cause of dyspnea. Given the ongoing melena and hemoglobin (Hb) drop, gastroenterology (GI) service was consulted and recommended EGD and colonoscopy. The cause of the bleeding was identified by the EGD as a metallic item that extended into the duodenum. The metallic object was found to be an IVC filter that was invading the duodenum and producing ulcers (Figure 1).

Vascular surgery was consulted, and the patient was taken immediately to the operation room (OR), where the IVC filter was removed. After removal of the foreign body from the duodenum CT abdomen was done and no further residual source for GI bleed was observed. The patient was discharged in hemodynamic stable condition.

## Discussion

IVC filter is a valuable tool for pulmonary embolism prevention in patients with venous thromboembolism [4]. In individuals who have a high risk of developing venous thromboembolism (VTE), prophylactic IVC filters may occasionally be implanted, particularly if anticoagulation is contraindicated. In patients where anticoagulation is not contraindicated studies have found no mortality advantage of IVC filters over anticoagulation, in fact, according to these studies, IVC filters are associated with a higher risk of complications including recurring deep vein thrombosis [5].

Complications of IVC filters can be classified into three categories. Procedure complications, post-procedure complications, and late complications [6]. Perforation falls in both the procedure and the late complications categories; it accounts for 20% of all adverse events reported on the manufacturer and user facility device experience (MAUDE) database [7]. Minimal penetration of the caval wall is required to anchor the filter to the vessel wall, it is considered pathological only when the limb protrudes more than 3mm beyond the caval wall [8]. Penetration is more common with conical filters [9]. Possible mechanisms of delayed perforation are thought to be IVC movement in conjunction with aortic pulsations and respiration [6].

The number of diagnosed cases with IVC filter perforation has increased over the past 40 years because of the availability of EGD, CT workups for abdominal complaints, and the availability of IVC filters (especially in the past 20 years) [10].

Perforations are usually asymptomatic. However, when the filter perforates neighboring structures, potentially serious clinical repercussions may emerge, perforations into the aorta, duodenum, portal vein, renal pelvis, pancreas, and diaphragm have been reported albeit being very rare [11-13]. In some patients, it may present with abdominal or back pain that they may experience for years, with or without hemorrhage [12]. Nausea, anorexia, intermittent constipation, and weight loss have also been reported [14].

Multiple case reports have discussed IVC filter perforation through the duodenum. Jehangir et al. [12] discussed the case of a 67-year-old woman with a lengthy history of abdominal pain and multiple DVTs with IVC filter insertion. Endoscopy demonstrated a thin metallic object embedded in the duodenal wall surrounded by erythema and edema. CT scan revealed the penetration of one prong of the IVC filter into the adjacent duodenal wall. Laparotomy was done with the removal of the filter and repair of the IVC and duodenum.

Another case was reported by Lee et al. [15] where a 63-year-old woman with a history of IVC filter insertion and mechanical thrombectomy for acute DVT presented with a duodenal foreign body found on EGD. X-ray and CT revealed penetration of the duodenum by the anterior strut of the filter and multiple caval penetrations. The filter was removed surgically with a good outcome.

Yamaguchi et al. [16] reported on a case of a 77-year-old woman who had a history of pulmonary embolism and IVC insertion and presented with chronic epigastric pain. CT showed the IVC filter penetrating the duodenal wall. EGD identified the filter leg penetrating the wall of the duodenum. The patient underwent laparotomy, and the filter was extracted. 

A case of a 33-year-old female was reported by Bae et al. [17] The patient suffered from epigastric pain with nausea and vomiting. She had a recent history of childbirth and DVT. IVC filter was inserted due to anticoagulation contraindication. CT and EGD demonstrated IVC filter penetration into the duodenum with signs of chronic progressive penetration. Laparotomy was done with filter extraction and duodenal repair.

## Conclusions

Perforation of an IVC filter into the duodenum is a rare but potentially dangerous complication that should be thought about in patients who have had a filter placed in the past and are having abdominal pain or gastrointestinal blood loss. Patients can have good results if they are carefully evaluated and treated with rapid vascular surgical intervention. Clinicians should carefully weigh the risks and benefits of putting in an IVC filter, taking into account factors unique to each patient and other ways to treat VTE.
